# Contrast-enhanced computed tomography plus gadolinium-ethoxybenzyl diethylenetriamine pentaacetic acid-enhanced magnetic resonance imaging for gross classification of hepatocellular carcinoma

**DOI:** 10.18632/oncotarget.15712

**Published:** 2017-02-24

**Authors:** Chuang Chen, Hui Zhao, Xu Fu, LuoShun Huang, Min Tang, XiaoPeng Yan, ShiQuan Sun, WenJun Jia, Liang Mao, Jiong Shi, Jun Chen, Jian He, Jin Zhu, YuDong Qiu

**Affiliations:** ^1^ Department of Hepatopancreatobiliary Surgery, Nanjing Drum Tower Hospital Clinical College of Nanjing Medical University, Nanjing 210008, Jiangsu, China; ^2^ Department of Hepatopancreatobiliary Surgery, Huai'an Hospital Affiliated to Xuzhou Medical University, Second People's Hospital of Huai'an City, Huai'an 223002, Jiangsu, China; ^3^ Department of Hepatopancreatobiliary Surgery, Nanjing Medical University Affiliated Wuxi Second Hospital, Wuxi 214001, Jiangsu, China; ^4^ Department of Hepatopancreatobiliary Surgery, The Affiliated Drum Tower Hospital of Nanjing University Medical School, Nanjing 210008, Jiangsu, China; ^5^ Department of Radiology, The Affiliated Drum Tower Hospital of Nanjing University Medical School, Nanjing 210008, Jiangsu, China; ^6^ Department of Pathology, The Affiliated Drum Tower Hospital of Nanjing University Medical School, Nanjing 210008, Jiangsu, China; ^7^ Key Laboratory of Antibody Technique of Ministry of Health, Nanjing Medical University, Nanjing 210029, Jiangsu, China; ^8^ Huadong medical Institute of Biotechniques, Nanjing 210029, Jiangsu, China

**Keywords:** hepatocellular carcinoma, CE-CT, EOB-MRI, gross classification, imaging

## Abstract

Accurate gross classification through imaging is critical for determination of hepatocellular carcinoma (HCC) patient prognoses and treatment strategies. The present retrospective study evaluated the utility of contrast-enhanced computed tomography (CE-CT) combined with gadolinium-ethoxybenzyl diethylenetriamine pentaacetic acid-enhanced magnetic resonance imaging (EOB-MRI) for diagnosis and classification of HCCs prior to surgery. Ninety-four surgically resected HCC nodules were classified as simple nodular (SN), SN with extranodular growth (SN-EG), confluent multinodular (CMN), or infiltrative (IF) types. SN-EG, CMN and IF samples were grouped as non-SN. The abilities of the two imaging modalities to differentiate non-SN from SN HCCs were assessed using the EOB-MRI hepatobiliary phase and CE-CT arterial, portal, and equilibrium phases. Areas under the ROC curves for non-SN diagnoses were 0.765 (95% confidence interval [CI]: 0.666–0.846) for CE-CT, 0.877 (95% CI: 0.793–0.936) for EOB-MRI, and 0.908 (95% CI: 0.830–0.958) for CE-CT plus EOB-MRI. Sensitivities, specificities, and accuracies with respect to identification of non-SN tumors of all sizes were 71.4%, 81.6%, and 75.5% for CE-CT; 96.4%, 78.9%, and 89.3% for EOB-MRI; and 98.2%, 84.2%, and 92.5% for CE-CT plus EOB-MRI. These results show that CE-CT combined with EOB-MRI offers a more accurate imaging evaluation for HCC gross classification than either modality alone.

## INTRODUCTION

Hepatocellular carcinoma (HCC) is the sixth most common malignancy, and the third most common cause of cancer-associated deaths worldwide [1]. Pathologically, HCCs exhibit morphological polymorphism according to their gross classification [2], which is closely related to prognosis, including post-treatment recurrence and patient survival rates [3–6]. This relationship has been validated in patients undergoing hepatic resection and living donor liver transplantation (LDLT) [7]. Microvascular invasion (MVI) and intrahepatic metastasis incidences were lower in single nodular (SN) type HCCs than in non-single nodular (non-SN) types [3–6, 8–11]. Patients with SN HCCs report better prognoses compared to those with non-SN types [5, 6, 8–11]. However, HCC gross classification is usually performed using resected specimens. Accurate gross classification via pre-treatment imaging is vital for improved HCC management [12].

Our previous studies showed that contrast-enhanced computed tomography (CE-CT) was a useful radiological technique for evaluating HCC gross classification [13]. Gadolinium ethoxybenzyl diethylenetriamine pentaacetic acid (Gd-EOB-DTPA) is a hepatocyte-specific magnetic resonance imaging (MRI) contrast agent with combined perfusion and hepatocyte-selective properties. In the hepatobiliary phase, HCCs without functioning hepatocytes usually show hypointensity compared with hyperintensity of the surrounding liver parenchyma owing to Gd-EOB-DTPA uptake by normal hepatocytes. This contrast agent reportedly enhances detection of small HCCs and predicts gross classification [14–16]. Contrast-enhanced MRI using Gd-EOB-DTPA (EOB-MRI) clearly distinguishes focal liver lesions from the surrounding hepatic parenchyma, and has the potential to precisely predict HCC gross classification [17].

An appropriate imaging modality is crucial for accurate HCC gross classification prior to surgery. In this study, we investigated CE-CT, EOB-MRI, and CE-CT plus EOB-MRI in HCC gross classification.

## RESULTS

### HCC pathological features

**Figure 1:**
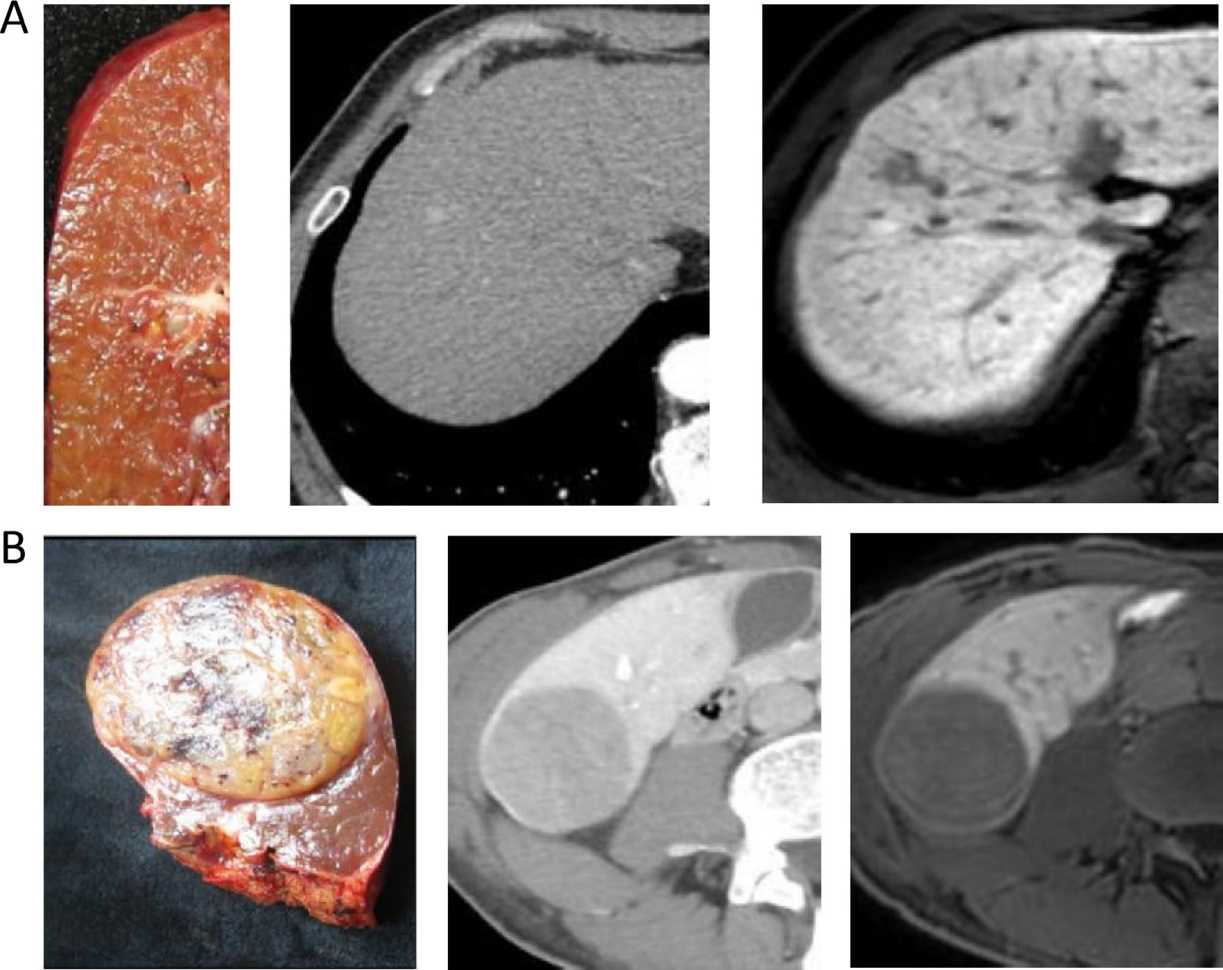
(**A**) A 66-year-old man with cirrhosis related to hepatitis B The gross classification was SN-IM based on pathological examination. Two images following specimen were arterial phase of CE-CT and hepatobiliary phase of EOB-MRI. (**B**) A 62-year-old man with cirrhosis related to hepatitis B. The gross classification was SN-DM based on pathological examination. Two images following specimen were equilibrium phase of CE-CT and hepatobiliary phase of EOB-MRI.

**Figure 2:**
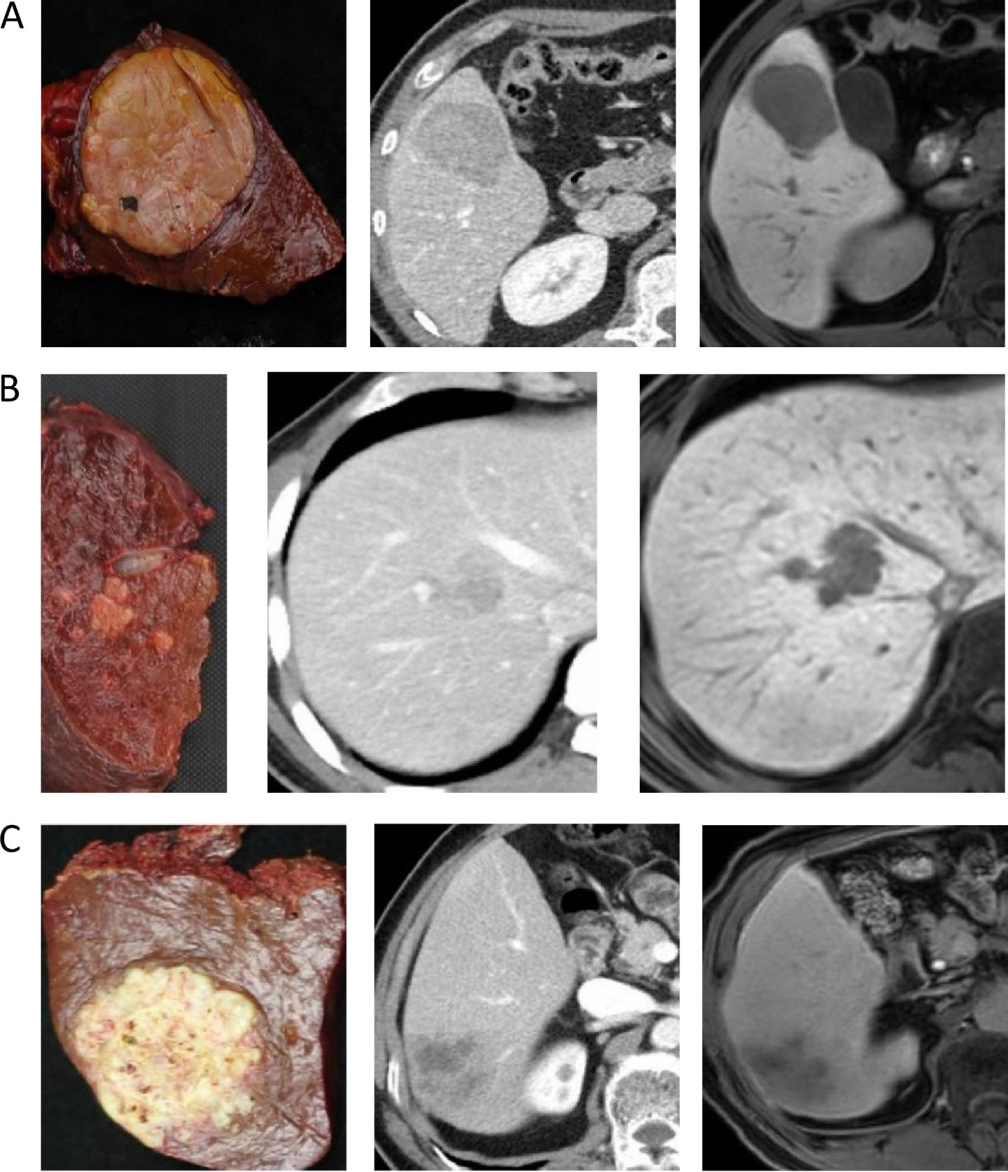
(**A**) A 42-year-old man with cirrhosis related to hepatitis B The gross classification was SN-EG based on pathological examination. Two images following specimen were arterial phase of CE-CT and hepatobiliary phase of EOB-MRI. (**B**) A 52-year-old man with cirrhosis related to hepatitis B. The gross classification was CMN based on pathological examination. Two images following specimen were portal phase of CE-CT and hepatobiliary phase of EOB-MRI. (**C**) A 64-year-old man with cirrhosis related to hepatitis B. The gross classification was IF based on pathological examination. Two images following specimen were portal phase of CE-CT and hepatobiliary phase of EOB-MRI.

Based on pathological macroscopic examination of the 94 HCCs, six tumors (6.4%) were classified as SN-IM (Figure 1A), 32 (34%) as SN-DM (Figure 1B), 26 (27.7%) as SN-EG (Figure 2A), 21 (22.3%) as CMN (Figure 2B), and nine (9.6%) as IF (Figure 2C). Therefore, 38 nodules (40.4%) were classified as SN and 56 (59.6%) as non-SN. Fifty-one (54.3%) tumors were ≤ 3 cm in size and 43 (45.7%) were > 3 cm. Mean sizes of all tumors, only SN types, and only non-SN types, were 3.7 ± 2.2 cm, 2.9 ± 1.8 cm, and 4.2 ± 2.3 cm, respectively.

### Relationships between gross classification and tumor factors

We investigated relationships between gross classification and tumor factors, including alpha-fetoprotein (AFP) level, tumor size, number of nodules, tumor cell differentiation, MVI, and intrahepatic metastasis. MVI incidence was 13.2% (5/38) for SN type tumors and 33.9% (19/56) for non-SN types (*P* = 0.01). Intrahepatic metastasis incidence was 0 (0 of 38) for SN types and 3.6% (2/56) for non-SN types (*P* = 0.241). Tumor size was associated with non-SN type HCC (*P* = 0.005) (Table 1). AFP, number of nodules, and tumor cell differentiation was similar between non-SN and SN types.

**Table 1:** Association between macroscopic findings and tumor factors

Variable	SN type (*n* = 38)	Non-SN type (*n* = 56)	*P* value
AFP (ng/ml) *	17.6 (2–1000)	22.8 (0.8–5722.1)	0.592
Maximum tumor size (≤ 3.0 cm/> 3.0 cm)	24/14	25/31	0.005
Number of tumor nodules (single/multiple)	30/8	49/7	0.269
Tumor cell differentiation (well or moderate/poor)	36/2	49/7	0.244
Microvascular invasion (present/absent)	5/33	19/37	0.010
Intrahepatic micrometastasis (present/absent)	0/38	2/54	0.241

^*^Data expressed as median (range).

### ROC curve analyses

**Figure 3:**
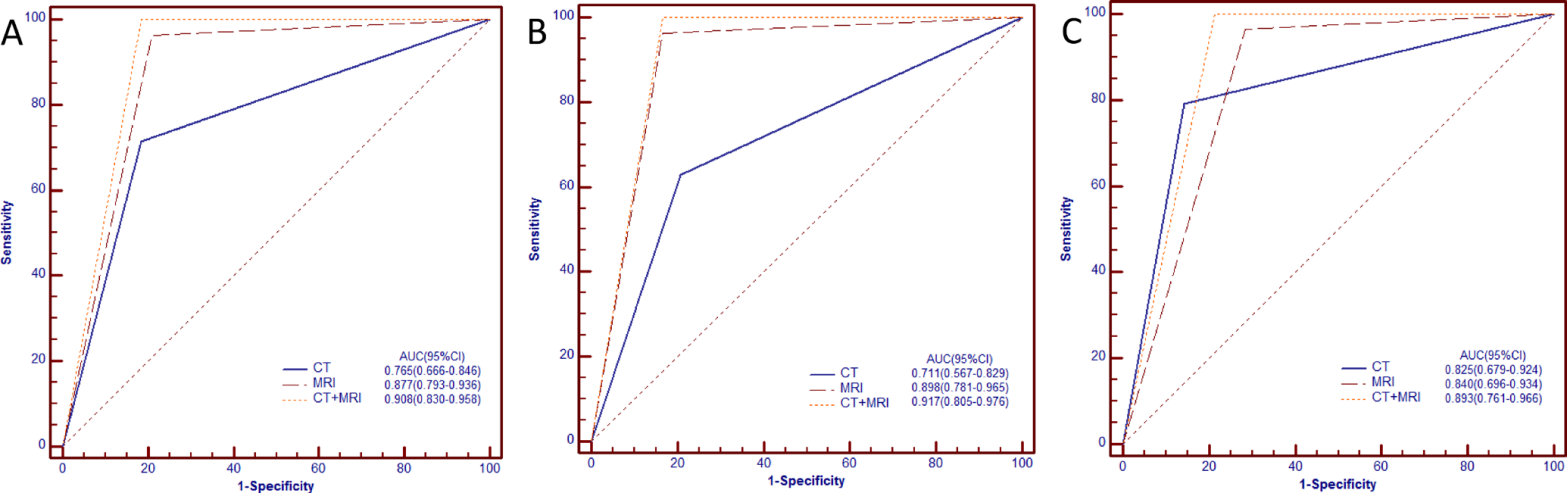
ROC curves of CE-CT, EOB-MRI and CE-CT plus EOB-MRI for diagnosing non-SN ((**A**), all sizes of HCCs; (**B**), Sizes of HCCs ≤ 3.0 cm; (**C**), Sizes of HCCs > 3.0 cm).

ROC curves for diagnosis of non-SN type HCC via CE-CT, EOB-MRI, and CE-CT plus EOB-MRI are shown in Figure 3. The AUC (95% confidence interval [CI]) for CE-CT, EOB-MRI, and CE-CT plus EOB-MRI for all tumor sizes were 0.765 (0.666–0.846), 0.877 (0.793–0.936), and 0.908 (0.830–0.958), respectively (Figure 3A). In HCCs ≤ 3 cm, AUC values for CE-CT, EOB-MRI, and CE-CT plus EOB-MRI were 0.711 (0.567–0.829), 0.898 (0.781–0.965), and 0.917 (0.805–0.976) (Figure 3B), and in HCCs > 3 cm were 0.825 (0.679–0.924), 0.840 (0.696–0.934), and 0.893 (0.761–0.966) (Figure 3C).

AUC values for CE-CT plus EOB-MRI, and EOB–MRI were higher than those for CE-CT for all tumor sizes (*P* = 0.006, 0.034, respectively) and HCCs ≥3 cm (*P* = 0.005, 0.016, respectively) (Table 2). In HCCs > 3 cm, CE-CT plus EOB-MRI AUC values were also higher than those for EOB–MRI (*P* = 0.182) and CE-CT (*P* = 0.361), but the difference was not significant.

**Table 2:** The AUC values of each modality and in combination for non-simple nodular type

	CT	MRI	CT+MRI	*P* values		
				CT vs MRI	CT vs CT+MRI	MRI vs CT+MRI
All sizes	0.765 (0.666–0.846)	0.877 (0.793–0.936)	0.908 (0.830–0.958)	0.034	0.006	0.088
HCCs ≤ 3 cm	0.711 (0.567–0.829)	0.898 (0.781–0.965)	0.917 (0.805–0.976)	0.016	0.005	0.317
HCCs > 3 cm	0.825 (0.679–0.924)	0.840 (0.696–0.934)	0.893 (0.761–0.966)	0.821	0.361	0.182

### Non-SN type tumor diagnostic abilities and misclassification incidences for all imaging modalities

Sensitivities, specificities, accuracies, PPVs, and NPVs for diagnosis of non-SN type tumors are shown in Table 3. Sensitivities for all tumor sizes, those ≤ 3 cm, and those > 3 cm were 71.4%, 62.9% and 79.3% for CE-CT, 96.4%, 96.3%, and 96.5% for EOB-MRI, and 98.2%, 96.3%, and 100% for CE-CT plus EOB-MRI, respectively. Specificities for all tumor sizes, those ≤ 3 cm, and those > 3 cm were 81.6%, 79.2%, and 85.7% for CE-CT, 78.9%, 83.3%, and 71.4% for EOB-MRI, and 84.2%, 87.5%, and 78.6% for CE-CT plus EOB-MRI, respectively. Accuracies for all tumor sizes, those ≤ 3 cm, and those > 3 cm were 75.5%, 70.6% and 81.4% for CE-CT, 89.3%, 90.2%, and 88.4% for EOB-MRI, and 92.5%, 92.2%, and 93% for CE-CT plus EOB-MRI, respectively. The sensitivities and accuracies of EOB-MRI, and CE-CT plus EOB-MRI were higher than those of CE-CT for all tumor sizes and those ≤ 3 cm, but in HCCs > 3 cm, the difference was not significant (Table 3). Nodule misclassification incidences were 24.5% (23/94) in CE-CT, 10.6% (10/94) in EOB-MRI, and 7.4% (7/94) in CE-CT plus EOB-MRI.

**Table 3:** Diagnostic ability of each modality and in combination for non-simple nodular type

	All size of HCCs (*n* = 94)						HCCs ≤ 3 cm (*n* = 51)						HCCs > 3 cm (*n* = 43)					
	CT	MRI	CT + MRI	*P_a_*	*P_b_*	*P_c_*	CT	MRI	CT + MRI	*P_a_*	*P_b_*	*P_c_*	CT	MRI	CT + MRI	*P_a_*	*P_b_*	*P_c_*
Sensitivity(%)	71.4	96.4	98.2	0.001	0.001	0.495	62.9	96.3	96.3	0.005	0.001	1.000	79.3	96.5	100	0.102	0.023	1.000
Specificity(%)	81.6	78.9	84.2	1.000	1.000	1.000	79.2	83.3	87.5	1.000	1.000	1.000	85.7	71.4	78.6	1.000	0.648	1.000
Accuracy(%)	75.5	89.3	92.5	0.020	0.002	0.612	70.6	90.2	92.2	0.023	0.010	1.000	81.4	88.4	93	0.549	0.195	0.713
PPV(%)	85.1	87.1	90.2	0.785	0.576	0.790	77.3	86.7	89.7	0.468	0.464	1.000	92	87.5	90.6	0.686	1.000	1.000
NPV(%)	65.9	93.7	96.9	0.005	0.001	0.492	65.5	95.2	95.4	0.016	0.003	1.000	66.7	90.9	100	0.202	0.058	1.000

PPV: positive predictive value, NPV: negative predictive value, *P_a_*: CT vs MRI, *P_b_*: CT vs CT + MRI, *P_c_*: MRI vs CT + MRI.

### Interobserver variability

Kappa values for CE-CT indicated moderate agreement for all tumor sizes (kappa value = 0.617), fair agreement for HCCs ≤ 3 cm (kappa value = 0.497), and good agreement for HCCs > 3 cm (kappa value = 0.802). Kappa values for EOB-MRI revealed good agreement for all tumor sizes and HCCs > 3 cm (kappa values = 0.802 and 0.776, respectively), and excellent agreement for HCCs ≤ 3 cm (kappa value=0.821). Kappa values for CE-CT plus EOB-MRI demonstrated good agreement for all tumor sizes, HCCs ≤ 3 cm and HCCs > 3 cm (kappa values = 0.730, 0.689, and 0.776, respectively).

## DISCUSSION

Factors such as intrahepatic metastasis, tumor size, vascular invasion, liver fibrosis, and cirrhosis influence tumor recurrence and patient survival following HCC resection [6, 18–26]. Minagawa, *et al*. reported that gross classification was an independent HCC prognostic factor in a nationwide study of Japanese patients [27], and Nakashima, *et al*., observed a higher incidence of intrahepatic metastasis and portal vein invasion in non-SN tumors compared to SN types [28]. Since non-SN type HCCs often invade within the tumor-bearing liver segment, systematized or anatomic hepatectomy is generally performed [29]. Gross classification of HCCs before treatment could significantly improve patient outcomes.

In this study, we assessed the abilities of CE-CT, EOB-MRI, and CE-CT plus EOB-MRI to diagnose non-SN HCC. The AUC values for diagnosis of non-SN type HCCs by CE-CT and EOB-MRI separately were > 0.7, which corresponds to a moderate diagnostic value. However, the values for CE-CT combined with EOB-MRI were > 0.9, corresponding to a higher diagnostic value. This difference was significant compared to CE-CT alone, but not to EOB-MRI. EOB-MRI alone was a superior diagnostic method compared to CE-CT for all tumor sizes, although as tumor diameter increased, the ability of CE-CT to diagnose non-SN type tumors improved. In HCCs > 3 cm, AUC values were > 0.8 for CE-CT, EOB-MRI, and CE-CT plus EOB-MRI. These results suggest that CE-CT combined with EOB-MRI improves the accuracy of HCC gross classification. This may be because EOB-MRI offers high soft tissue contrast and superior spatial resolution, as well as hepatobiliary phase images. The EOB-MRI hepatobiliary phase may improve signal contrast between hypointense HCCs and the surrounding enhancing liver parenchyma, and thus tumor margins are clearly displayed [30].

In HCCs ≤ 3 cm, EOB-MRI diagnostic sensitivity and accuracy values in our study were similar to those reported by Toshifumi, *et al*. [31] (96.4% vs. 96.9% and 89.3% vs. 83.1%, respectively). The diagnostic accuracy of CE-CT was comparable with that reported by Toshifumi, *et al*. (70.6% vs. 70.4%) [31]. Simultaneous analyses of arterial, venous and equilibrium phases of thin-sliced CE-CT, as well as recent advances in imaging techniques, may contribute to the higher diagnostic accuracy observed in our study. Additionally, tumor specimens in our study were cut similar to the axial image section, and this may have helped improve CE-CT diagnostic accuracy.

Each imaging modality studied has limitations. For hypovascular HCCs, CE-CT cannot show tumor margins clearly. Since microvascular invasion or portal vein tumor thrombosis is present in some tumors, peritumoural liver parenchyma could reduce enhancement on hepatobiliary phase images, resulting in decreased tumor-liver contrast [32]. Furthermore, 10–20% of HCCs remain isointense or even hyperintense during the hepatocellular phase, which correlates with expression of organic anion-transporting polypeptides (OTAPs) in tumor cells [33, 34]. It is challenging to clearly display tumor margins on EOB-MRI hepatobiliary phase images, and this can lead to misdiagnosis. In the current study, seven nodules were misclassified via CE-CT plus EOB-MRI, as a result of misdiagnosis of SN type tumors as non-SN, and these misclassified nodules were treated with anatomical hepatectomy. However, most nodules misclassified in this study resulted from misdiagnosis of non-SN type tumors as SN via CE-CT, especially in HCCs ≤ 3 cm. Similar results were reported by Tomoki, *et al*. [12]. This problem likely occurred because smaller tumor sizes result in lower contrast and spatial resolution, and because of poor contrast-enhancement, particularly in CE-CT. In the present study, HCC gross classification via CE-CT plus EOB-MRI exhibited higher accuracy and sensitivity compared to either modality alone.

The number of patients with MVI was greater in non-SN type (33.9%) than in SN type HCCs (13.2%, *P* = 0.01). Non-SN classification is an MVI predictor in HCCs and contributes to poor patient prognosis post-hepatic resection or transplantation [7, 22, 28]. Anatomic hepatectomy or partial hepatic resection with a wide tumor margin is recommended to eradicate MVI [35, 36]. Misclassification of non-SN type tumors as SN might negatively impact patient survival due to differences in treatments or surgical procedures resulting from misdiagnosis.

The present study has some limitations. First, ours was a single-center retrospective study with a relatively small patient population. Further studies with a larger number of patients are needed. Second, 92 of the study patients were Child-Pugh class A, which suggests they had good liver conditions. Enrolling more patients with poor liver conditions (Child-Pugh class B or C) might decrease the ability of EOB-MRI plus CE-CT to distinguish non-SN type tumors. Third, this study lacked prognosis information for a portion of the enrolled patients. It will essential to clarify tumor recurrence and patient survival rates for HCCs diagnosed as non-SN via EOB-MRI and/or CE-CT in the future.

In conclusion, accurate gross classification through imaging is critical for determination of HCC patient prognoses and appropriate treatment strategies. HCC diagnosis via CE-CT plus EOB-MRI might provide a more accurate imaging assessment for HCC gross classification.

## MATERIALS AND METHODS

### Patients

Study protocols were approved by our Institutional Review Board. Patient informed consent was waived due to the retrospective nature of the study. From July 2014 to May 2016, 153 consecutively registered patients with HCCs were subjected to curative hepatectomy at our institution. We based HCC diagnoses on postoperative pathology. Patients were included according to the following criteria: (1) gross specimen photographs could be reviewed; (2) no preoperative therapy was provided, such as radiofrequency or microwave ablation, transcatheter arterial chemoembolization (TACE), or molecular targeted therapy; (3) both CE-CT and EOB-MRI were preoperatively performed. Ninety-four patients meeting these criteria were enrolled in our study (Table 4). Another 59 patients were excluded for the following reasons: tumors with other cell types, such as cholangiocellular carcinoma (16 cases) or metastatic liver cancer (22 cases), and incomplete clinical data (21 cases). In patients with multiple HCCs, a major tumor was analyzed.

**Table 4:** Patient and hepatocellular carcinoma characteristics

Variables	Value (*n* = 94)
Age (years)*	58.1 ± 10.3
Sex	
Male	84
Female	10
Etiology	
Hepatitis B infection	90
Hepatitis C infection	2
Co-infection	1
Hepatitis negative	1
Child-Pugh grade	
A	92
B	2
MELD score^#^	7 (6–18)
ICG-R15	5.9 ± 3.1
AFP(ng/ml) ^#^	17.6 (0.8–5722.1)
Platelet count (×10^9^ g/L)*	132 ± 54.9
HKLC staging (I/II/III/IV/V)	70/17/3/4/0
Tumor number	
Single nodule	79
Two nodules	11
Three nodules	4
Tumor size (cm) *	3.7 ± 2.2
≤ 3 cm	51 (2.2 ± 0.6)
> 3 cm	43 (5.5 ± 1.9)
SN^†^/non-SN^¶^ (cm)	2.9 ± 1.8/4.2 ± 2.3
Pathological gross classification (SN/non-SN)	38/56
SN-IM	6
SN-DM	32
SN-EG	26
CMN	21
IF	9
Tumor cell differentiation	
well-differentiated	10
moderate-differentiated	75
poorly-differentiated	9
Microvascular invasion (present/absent)	24/70
Intrahepatic micrometastasis (present/absent)	2/92

HKLC: Hong Kong Liver Cancer, ICG-R15: indocyanine green retention rate at 15 min, MELD: model for end-stage liver disease, *, data expressed as means (standard deviation), ^#^, data expressed as median (range), ^†^, SN defined as both SN-IM and SN-DM, ^¶^, Non-SN defined as SN-EG,CMN and IF.

The 94 patients included 84 men (89.4%) and 10 women (10.6%), with mean age 58.1 ± 10.3 years (range 35–78 years). Numbers of patients with positive hepatitis B surface antigen, positive hepatitis C virus antibody, positive for both, or negative for both were 90 (95.7%), 2 (2.1%), 1 (1.1%) and 1 (1.1%), respectively. The median AF*P* value was 17.6 (0.8–5722.1) ng/ml. The Child-Pugh classification system was used to evaluate liver function, with 92 patients classified as A and two as B [37].

### Imaging methods

We performed MRI via a 3.0 T MRI system (Ingenia 3.0 T, Philips Healthcare). For gadoxetic acid-enhanced MRI, we obtained unenhanced and triple-arterial-phase (with a fixed 18-s scanning delay, each of these data sets lasted only 8 s), portal-phase (60 s), late-phase (3 min), and 20-min hepatobiliary phase images using a T1-weighted 3D turbo-field-echo (TFE) sequence with a multiecho Dixon fat-water separation technique (TR, 3.6 ms; TE1, 1.25 ms; TE2, 2.3 ms; flip angle, 10°; matrix size, 300 × 250; field of view, 400 mm (RL) × 350 mm (AP); section thickness, 2 mm; intersection gap, 0 mm). 10 mL of Gd-EOB-DTPA (Bayer Schering Pharma, Berlin, Germany) was given as an intravenous bolus (1.5 mL/s) and flushed with 20 ml of sterile saline solution from the antecubital vein through a power injector. T2-weighted and diffusion-weighted images were taken after gadoxetic acid administration. HCC gross classification via EOB-MRI was performed mainly using the hepatobiliary phase.

All patients were examined with multidetector spiral CT (LightSpeed VCT; GE Healthcare) in pre-contrast enhanced and multiphase contrast-enhancement scans with a slice thickness of 1.25 mm. Hepatic arterial phase, portal venous phase, and equilibrium phase images were obtained at 30, 60 and 180 s with 2 mL/kg bodyweight contrast media (Omnipaque 350 mgI/mL; GE Healthcare) injected through the antecubital vein (4 mL/s). Other abdominal CT scan parameters were as follows: tube voltage 120 kVp, tube current 240 mA, rotation time 0.6 s, helical pitch 1.375, field of view 35–40 cm, and matrix 512 × 512. HCC gross classification via CE-CT was based on the arterial, portal and equilibrium phases.

### Pathological assessment of gross classification

**Figure 4:**
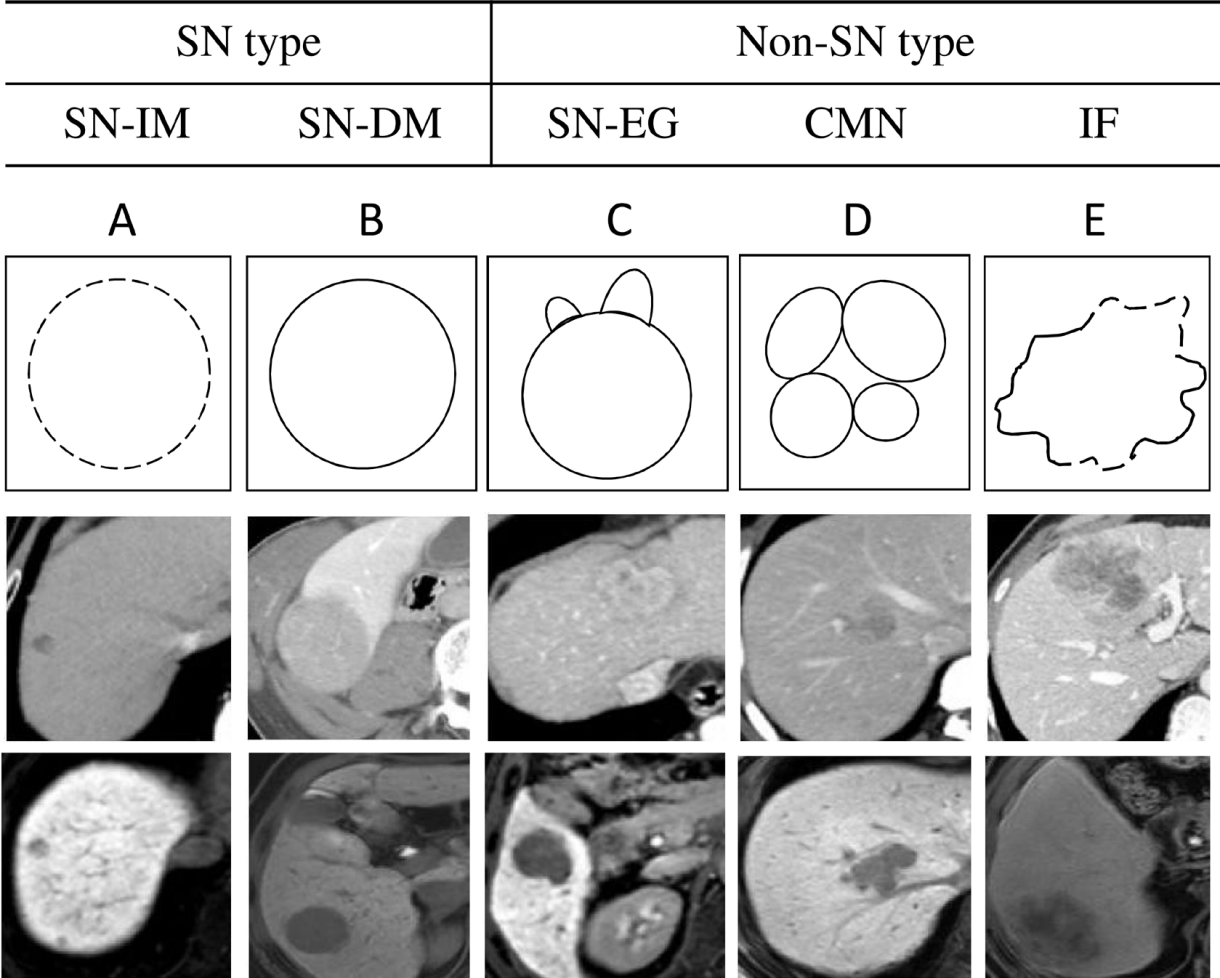
Summaries of the five gross classification of hepatocellular carcinoma Top schemas show the schematic diagram of the five gross classification as follows: (**A**) SN-IM, (**B**) SN-DM, (**C**) SN-EG, (**D**) CMN, and (**E**) IF. Middle images show the arterial phase, portal phase or equilibrium phase of CE-CT. Bottom images show the hepatobiliary phase of EOB-MRI.

A section of each resected tumor specimen was collected similarly to the section used for axial CT and MRI before immediate photographing and fixation in 10% formalin. In the current study, these photographs were used to retrospectively assess HCC gross classification. In light of the General Rules of the Clinical and Pathological Study of Primary Liver Cancer in Japan [2], two experienced pathologists and two surgeons divided pathological macroscopic findings from tumor specimens in this study into five types: (1) simple nodular with indistinct margin (SN-IM), (2) simple nodular with distinct margin (SN-DM), (3) simple nodular with extranodular growth (SN-EG), (4) confluent multinodular (CMN), and (5) infiltrative (IF) (Figure 4). Macroscopic evaluations were conducted independently by one hepatologist (Hui Zhao, with 11 years of experience) and one pathologist specializing in hepatology (Jiong Shi, with eight years of experience); both evaluators were blinded to patients data. The readers independently made their classifications and in cases of disagreement a consensus was reached after discussion.

### Imaging assessment of gross classification

Through retrospective review of both CE-CT and EOB-MRI images, we categorized 94 nodules into five gross types, including SN-IM, SN-DM, SN-EG, (tumors were round with extranodular growth), CMN (tumors were lobulated with multiple nodular lesions), and IF (tumors were lobulated with an irregular margin). We then divided these five gross types into two groups: simple nodular (SN), including SN-IM and SN-DM, and non-simple nodular (non-SN), including SN-EG, CMN, and IF, because the latter had higher malignant potential than the former. Preoperative CE-CT and EOB-MRI images were reviewed by one hepatologist (Xu Fu, with four years of experience) and one radiologist specializing in hepatology (Min Tang, with 10 years of experience) independently; both evaluators were blinded to patient data. They independently evaluated hepatobiliary phase EOB-MRI axial images, as well as CE-CT arterial, venous and equilibrium phase axial images to predict the gross classification of nodules. The readers independently made their classifications and in cases of disagreement a consensus was reached after discussion.

### Statistical analysis

Data were presented as means ± standard deviation or median (range). The Mann-Whitney *U* test was performed to compare continuous variables, and Fisher's exact test compared categorical variables. The receiver operating characteristic (ROC) curve was applied to evaluate the diagnostic performance of CE-CT, EOB-MRI and both combined [38]. An area under the ROC curve (AUC) of > 0.90 corresponded to high accuracy, 0.70–0.90 indicated moderate accuracy, and 0.50–0.70 low accuracy [39]. The Chi squared test compared sensitivities, specificities, accuracies, positive predictive values (PPV), and negative predictive values (NPV). For the evaluation of interobserver variability with respect to classified imaging findings, kappa statistics was conducted. Agreement was considered to be almost perfect at a kappa value of 1.00–0.81; good at 0.80–0.61; moderate at 0.60–0.41; fair at 0.40–0.21; and poor at < 0.21 [40]. All data were analyzed using SPSS19.0 statistical software (SPSS, Chicago, IL, USA) and MedCalc11.4.2 software (Ostend, Belgium). *P* < 0.05 indicated statistical significance.
